# Supplementary material for: Solid Dispersion of Resveratrol Supported on Magnesium DiHydroxide (Resv@MDH) Microparticles Improves Oral Bioavailability
Source: Nutrients. 2018 Dec 5;10(12):1925. doi: 10.3390/nu10121925 (PMC6315708; doi:10.3390/nu10121925)
Supplement: Supplementary file 1 [file nutrients-10-01925-s001.pdf]

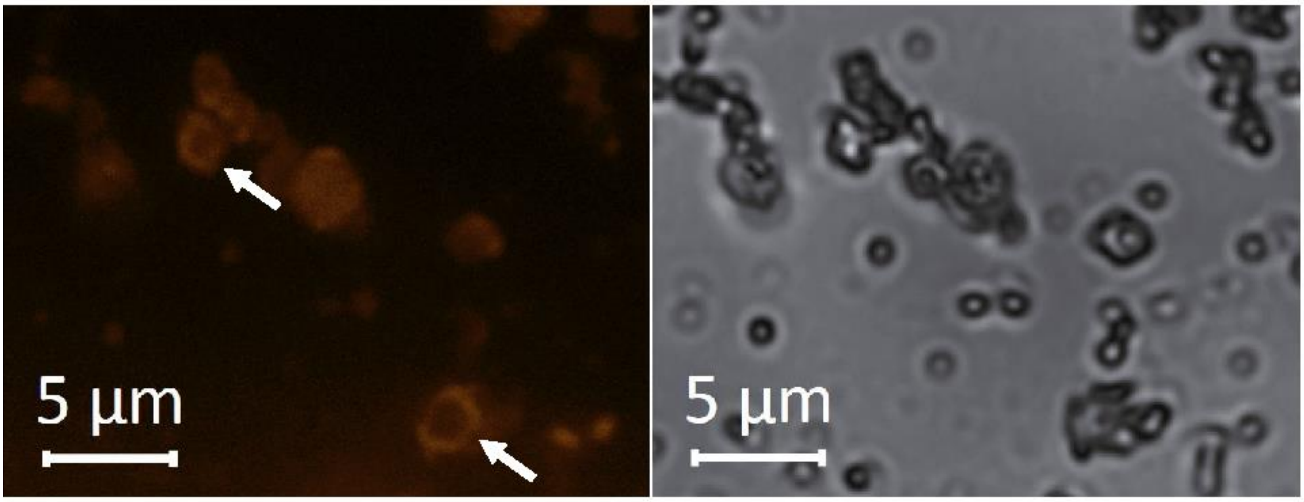

Supplemental Figure 1: Image of RESV@MDH powder dispersed in glycerol under different eccitation sources. (**A**) Rohdamine fluorescence filter (**B**) brightfield; Note: white arrow indicates the fluorescent shell of resveratrol around the core of magnesium diihydroxide.

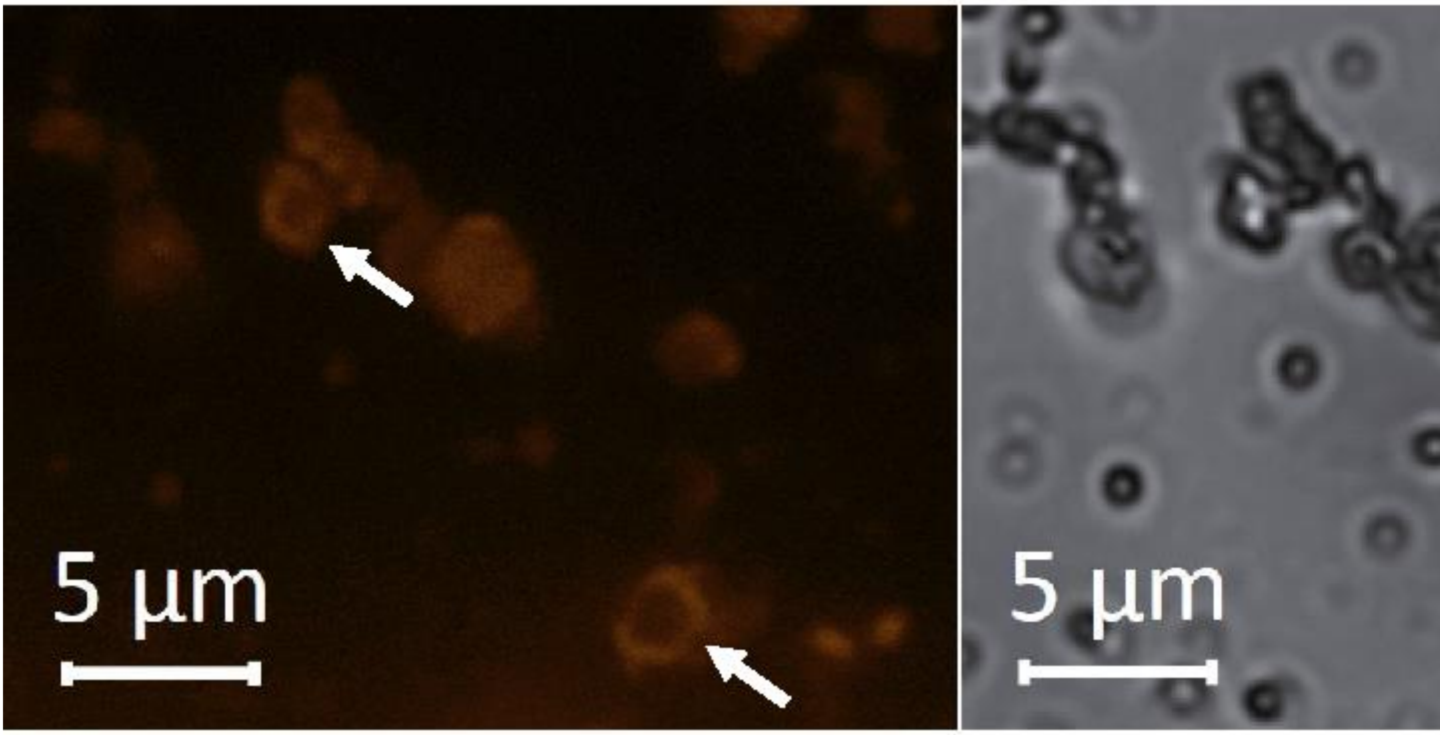
5 µm
5 µm
